# Evaluation of Semen Quality of Jeju Black Cattle (JBC) to Select Bulls Optimal for Breeding and Establish Freezing Conditions Suitable for JBC Sperm

**DOI:** 10.3390/ani12050535

**Published:** 2022-02-22

**Authors:** Jae-Wook Yoon, Seung-Eun Lee, Won-Jae Kim, Dae-Cheol Kim, Cheol-Ho Hyun, Shin-Ji Lee, Hyo-Jin Park, So-Hee Kim, Seung-Hwan Oh, Do-Geon Lee, Da-Bin Pyeon, Eun-Young Kim, Se-Pill Park

**Affiliations:** 1 Faculty of Biotechnology, College of Applied Life Sciences, Jeju National University, 102 Jejudaehak-ro, Jeju-si 63243, Korea; jwyoon9207@jejunu.ac.kr (J.-W.Y.); foxwoman20@jejunu.ac.kr (S.-E.L.); wonj1140@naver.com (W.-J.K.); pulbit1@jejunu.ac.kr (H.-J.P.); kshz1018@naver.com (S.-H.K.); kingsh2088@gmail.com (S.-H.O.); genemeditation@gmail.com (D.-G.L.); pyun03@gmail.com (D.-B.P.); jlokey@daum.net (E.-Y.K.); 2 Stem Cell Research Center, Jeju National University, 102 Jejudaehak-ro, Jeju-si 63243, Korea; 3 Jeju Special Self-Governing Province Livestock Promotion Agency, 13 Sinbimaeul, Jeju-si 63078, Korea; kdc5779@korea.kr (D.-C.K.); hch2423@korea.kr (C.-H.H.); sj99@korea.kr (S.-J.L.); 4 Mirae Cell Bio, 1502 ISBIZ Tower, 147 Seongsui-ro, Seongdong-gu, Seoul 04795, Korea

**Keywords:** Jeju black cattle, sperm, cryopreservation, motility, vitality, morphology

## Abstract

**Simple Summary:**

Jeju black cattle, a type of native Korean cattle characterized by black fur covering the entire body, inhabit Jeju Special Self-Governing Province, a World Natural Heritage Site. Although this breed was state designated as a natural monument in 2013 due to its characteristics and genetic traits, it is on the verge of extinction and thus there is a need to preserve this breed and further improve its traits. Therefore, we evaluated sperm motility, vitality, and morphology, which have long been considered good predictors of fertility in the absence of female infertility factors. Our findings showed that the semen of the JBC-A bull was superior to the semen of four other JBC bulls. Due to the aging of the population of JBC breeding bulls, strategies should be devised to improve sperm production in vivo.

**Abstract:**

To optimize the reproduction of Jeju black cattle (JBC), freezing conditions for sperm were established and sperm motility, vitality, morphology, and fertility were evaluated to select the optimal bull for breeding. Semen samples from five JBC bulls were individually mixed with freezing medium at a final concentration of 1 × 108 sperm/mL and frozen in liquid nitrogen vapor at a height of 3 or 7 cm (referred to as 3 cm sperm and 7 cm sperm, respectively). When the freezing conditions were compared, the motility of 7 cm sperm was significantly higher than that of 3 cm sperm for the JBC-A bull. The motility, curvilinear velocity, straight-line velocity, and average path velocity of fresh and frozen–thawed sperm were the highest for the JBC-A bull. The vitalities of fresh and frozen–thawed sperm were the highest for the JBC-A/E and JBC-A bulls, respectively. The percentage of normal cells in fresh sperm was the highest for the JBC-D bull. The rates of the normal formation of two pronuclei and total sperm penetration were the highest in zygotes fertilized with sperm from the JBC-A bull. The sperm from the JBC-A bull had superior qualities and are thus the most appropriate choice for the preservation and reproduction of these endangered cattle.

## 1. Introduction

Modern innovations in animal husbandry allow the establishment of animal genetic resources. Farmers depend on these innovations to obtain better-quality domestic animals. However, genetic materials from animals of economic merit, wild animals, and endangered species may be lost due to unexpected reproductive failure or death. Efforts are thus needed to avoid the loss of these genetic materials.

Four cattle breeds distinguished by coat color patterns are native to Korea, namely, a brown or red breed (Hanwoo), a brown breed with black stripes (Korean brindle cattle, also called Chick-so), and two black breeds distinguished by their localities, with Korean black cattle living on the mainland and Jeju black cattle (JBC) on Jeju Island. There are an estimated 2.9 million Hanwoo cattle in Korea, whereas there are small populations of the other three breeds, especially JBC [1]. JBC, which are characterized by black fur covering the entire body, live in Jeju Special Self-Governing Province, a World Natural Heritage Site. The qualities and genetic uniqueness of this breed resulted in its designation as a protected, state-designated national monument in 2013. However, this breed is endangered, with approximately 1400 animals remaining in Jeju Special Self-Governing Province. Elite breeding bulls are therefore needed to preserve this breed and to further improve its traits.

Male reproductive capacity is a major factor in mammalian reproduction and depends on the quality of germ cells produced by male animals. Sperm quality can be determined by measuring three important parameters: motility, morphology, and vitality. Vitality is the proportion of live spermatozoa in a sample, as determined by the evaluation of membrane integrity [2]. Differences in the fertility of males used for artificial insemination may result from differences in sperm morphology [3,4] because a normal morphological structure is generally required for sperm to reach the ovum and activate oocytes [5]. Motility is also important. Indeed, the injection of sperm with different levels of motility into females results in a backflow of poorly motile sperm after 15 min [5,6]. Therefore, semen analysis is the most commonly performed procedure to evaluate male fertility of humans and animals [7,8,9,10]. The objective of this study was to select the optimal JBC bulls for breeding this endangered breed by evaluating the sperm motility, vitality, and morphology of five JBC bulls following the establishment of suitable freezing conditions for their semen.

## 2. Materials and Methods

### 2.1. Chemicals and Reagents

All chemicals and reagents were purchased from Sigma (St. Louis, MO, USA) unless stated otherwise.

### 2.2. Ethics Statement and Animals

All animal studies were carried out in strict accordance with the recommendations in the Guide for the JBC Conservation Management Plan at the Jeju Special Self-Governing Province Livestock Promotion Agency and Guide for the Care and Use Committee (IACUC) of the Jeju National University (No. 2022-0010). Semen samples were collected from 38 ejaculates (6–10 samples per bull) from 5 JBC bulls using an artificial vagina in the morning hours every 2 weeks for 20 weeks. Semen from clinically healthy JBC bulls aged 2 to 7 years (*n* = 5) were used. These animals were bred at the Livestock Promotion Agency in Jeju Special Self-Governing Province, Korea, with compound feed and roughage feed twice a day. The Livestock Promotion Agency distributes frozen semen to farmers; therefore, this study focused on frozen semen, and data for fresh semen were collected as a control to determine whether freezing should be performed.

### 2.3. Freezing and Thawing Process

Semen samples were mixed with freezing medium (20% egg yolk and 20% Triladyl) to yield a concentration of 1 × 108 sperm cells/mL. Sperm was cooled by placing 0.5 mL plastic straws filled with diluted semen samples in a refrigerator at 4 °C for 2 h. The straws were placed 3 or 7 cm (referred to as 3 cm sperm and 7 cm sperm, respectively) over liquid nitrogen (LN2) vapor for 10 min and then directly plunged into LN2 for storage. Straws were thawed by immersing them in a 37 °C water bath for 30 s.

### 2.4. Sperm Analysis Imaging System

Sperm motility was assessed using the Sperm Analysis Imaging System (SAIS Plus, Medical Supply Co. Ltd., Busan, Korea). Semen samples (10 μL) were placed in a pre-warmed makler counting chamber and sperm motility characteristics were analyzed. At least 200 sperm from 5 fields were counted per reading. The software used for analysis was SAIS Advanced v.2008. Sperm motility (motility, %) was assessed by measuring the percentage of sperm cells showing forward progress. Other sperm parameters measured included curvilinear velocity (VCL, μm/s), straight-line velocity (VSL, μm/s), average path velocity (VAP, μm/s), linearity (LIN, %), straightness (STR, %), beat cross frequency (BCF, Hz), and amplitude of lateral head displacement (ALH, μm/s). Semen samples from five JBC bulls were analyzed 6–10 times each.

### 2.5. Analysis of Sperm Vitality

**Figure 1:**
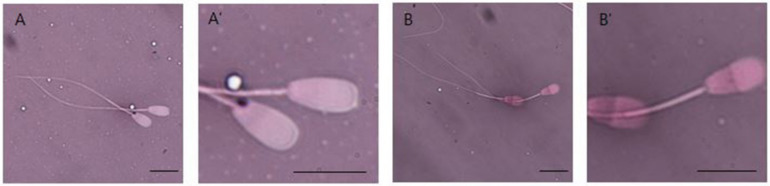
Eosin–nigrosin smear observed in bright field optics. Spermatozoa with red and dark pink heads were considered dead, whereas spermatozoa with white and light pink heads were considered alive. (**A**,**A′**): live sperm; (**B**,**B′**): dead sperm. Scale bars, 5 µm.

The vitality of spermatozoa was assessed by the eosin–nigrosin (Junsei Chemical Co., Chuo-ku, Tokyo, Japan) staining technique, with some modifications. This method was based on the degree of membrane permeability of dead spermatozoa, the heads of which are colored pink or red, whereas the low permeability of live gametes excludes eosin, with their heads remaining whitish. Briefly, a small amount of 2% eosin solution was mixed with a similar volume (10 μL) of each sample. Following incubation for 60 s, 40 μL of 10% nigrosin was added, the solution was mixed, and 5 μL of each stained sample was smeared onto a clean slide. Each slide was dried by placement on a warm (37 °C) plate, followed by an examination of 200 spermatozoa per slide at a magnification of 1000× with a bright field microscope. Sperm stained red or pink were classified as dead, except for sperm with light pink coloration of the neck region, which were classified as live (Figure 1) [11]. Semen samples from five JBC bulls were analyzed 6–10 times each.

### 2.6. Analysis of Sperm Morphology

**Figure 2:**
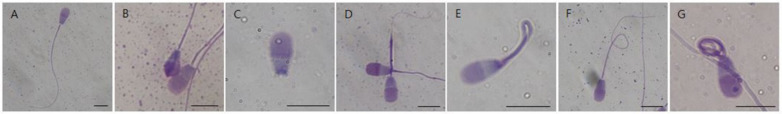
Morphology of bovine sperm determined by Diff-Quik staining. (**A**): Normal; (**B**): abnormal head; (**C**): detached head; (**D**): abnormal midpiece; (**E**): bent midpiece; (**F**): bent tail; and (**G**): coiled tail. Scale bars, 5 µm.

Sperm morphology and staining features were assessed using Diff-Quik kits (Sysmex, Kobe Hyogo, Japan), which contain a fixative (methanol), an anionic/acidic dye (eosin) that stains positively charged/basic proteins red, and a cationic dye (methylene blue and its derivatives) that stains nuclei and negatively charged molecules blue. Briefly, 5 µL of a sperm suspension was smeared onto each slide, covered with a coverslip, and allowed to dry on a warm plate (40 °C). The slides were immersed in methanol for 60 s, allowed to air dry, immersed sequentially for 60 s each in each kit solution, and rapidly dipped in water to remove excess dye. The slides were allowed to air dry, and 200 spermatozoa on each slide were observed at a magnification of 1000× with a bright field microscope. Based on their morphological characteristics, the spermatozoa were classified into seven categories: normal, abnormal head, detached head, abnormal midpiece, bent midpiece, bent tail, and coiled tail (Figure 2). Semen samples from five JBC bulls were analyzed 6–10 times each.

### 2.7. Oocyte Preparation and In Vitro Maturation (IVM)

Bovine ovaries were collected from a local slaughterhouse, immersed in 0.9% saline supplemented with 75 μg/mL penicillin G and 50 μg/mL streptomycin sulfate, and transported to the laboratory within 2 h at 30–33 °C. Generally, there are many cows over 10 years old. Cumulus–oocyte complexes (COCs) were aspirated from follicles with a diameter of 2–6 mm using an 18-gauge needle and a disposable 10 mL syringe. COCs were washed three times in tissue culture medium (TCM)-199–HEPES containing 0.1% (*w*/*v*) bovine serum albumin (BSA). Sets of ten COCs were matured in 50 μL droplets of TCM-199 (Gibco, Grand Island, NY, USA) supplemented with 10% fetal bovine serum, 0.2 mM sodium pyruvate, 0.5 µg/mL follicle-stimulating hormone, 1 μg/mL estradiol-17β, and 0.1 μg/mL gentamycin under mineral oil for 21 h at 38.8 °C in air containing 5% CO_2_.

### 2.8. In Vitro Fertilization (IVF)

At 21 h after IVM, COCs were transferred to TL-STOCK medium (114 mM NaCl, 3.2 mM KCl, 25 mM NaHCO_3_, 0.4 mM NaH_2_PO_4_·H_2_O, 2 mM CaCl_2_·2H_2_O, 0.5 mM MgCl_2_·6H_2_O, 10 mM Na-lactate, and 0.2 mM Na-pyruvate) containing 6 mg/mL fatty acid-free (FAF) BSA and inseminated with 2 μL of highly motile sperm (2.5 × 107 spermatozoa/mL), recovered from the thawed semen of JBC bulls. Two microliters each of heparin (2 µg/mL) and PHE (18.2 M penicillamine, 9.1 M hypotaurine, and 1.8 M epinephrine) was added to the 44 µL IVF drop. Egg yolk was removed by slowly adding thawed semen to 500 µL of 20% Triladyl solution (Triladyl: distilled water = 1:4) and incubating the sample at room temperature for 5 min. A 1 mL aliquot of sperm in 20% Triladyl solution was gently layered on top of the Sperm Filter (80% and 40% layered; 1 mL) and incubated at 38 °C for 5 min. The suspension was centrifuged at 200× *g* (2000 rpm) for 5 min, the supernatant was aspirated, and the pelleted sperm were counted using a hemocytometer and diluted to 2.5 × 107 cells/mL by adding SP-TALP (100 mM NaCl, 3.1 mM KCl, 25 mM NaHCO_3_, 10 mM HEPES, 0.29 mM NaH_2_PO_4_·H_2_O, 2.1 mM CaCl_2_·2H_2_O, 0.4 mM MgCl_2_·6H_2_O, 21.6 mM Na-lactate, and 1 mM Na-pyruvate) containing 6 mg/mL FAF-BSA.

### 2.9. Evaluation of Sperm Penetration of Oocytes In Vitro

Sperm penetration was defined as the presence of two pronuclei (2 PN) and/or a sperm head in an oocyte at 18 h after IVF. To remove cumulus cells, IVF embryos were treated with TL-HEPES containing 0.1% hyaluronidase. Denuded embryos were washed with TL-HEPES and fixed for 2–3 min in 2% formaldehyde. Fixed embryos were stained with 25 μg/mL bisbenzimide (Hoechst 33258) for 10 min, washed three times, mounted onto glass slides, and examined by fluorescence microscopy at a magnification of ×200. Two JBC bulls were analyzed three times each.

### 2.10. Statistical Analysis

Significant differences among bulls were analyzed using paired Tukey’s multiple range tests. Data were analyzed using the general linear model procedure within the Statistical Analysis System (SAS User’s Guide, 1985, Statistical Analysis System Inc., Cary, NC, USA). *p* <  0.05 was considered statistically significant.

## 3. Results

### 3.1. JBC Sperm Motility

**Figure 3:**
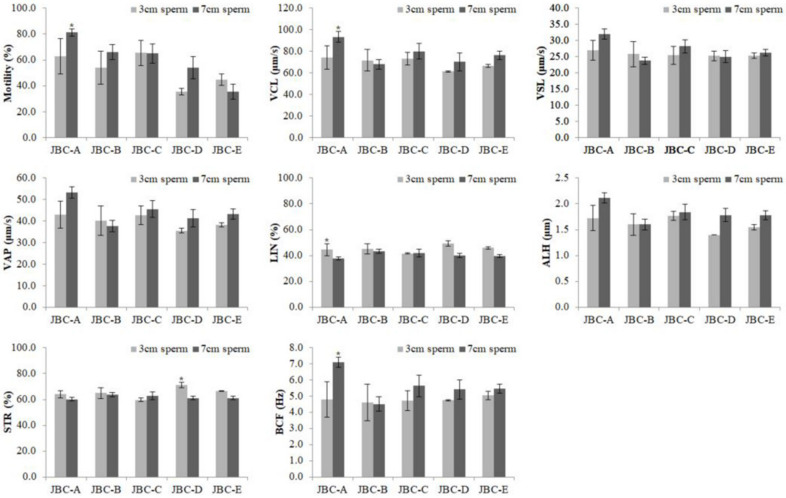
Motility parameters of frozen–thawed 3 and 7 cm sperm from individual Jeju black cattle (JBC) bulls, as evaluated by SAIS. Data shown are the mean ± SEM of three independent replicates per group. * *p* < 0.05. VCL, curvilinear velocity; VSL, straight-line velocity; VAP, average path velocity; LIN, linearity; ALH, amplitude of lateral head displacement; STR, straightness; BCF, beat cross frequency.

The motility of sperm from individual JBC bulls was analyzed using the SAIS. To check the effect of the height at which sperm were exposed to LN2 vapor, the optimal freezing conditions were determined at a height of 3 or 7 cm (Figure 3). The motility of 3 cm sperm was significantly lower than that of 7 cm sperm for the JBC-A bull (*p* < 0.05). However, the motility of 3 and 7 cm sperm did not significantly differ for the other four bulls (JBC-B, JBC-C, JBC-D, and JBC-E). Therefore, we compared 7 cm sperm in subsequent experiments.

The motility of fresh sperm (Table 1) ranged from 96.1% to 98.7%. The sperm from the JBC-A bull had significantly higher motility than the sperm from the JBC-C bull (*p* < 0.05), but sperm motility did not significantly differ between the JBC-A bull and the three other bulls. VCL, VSL, and VAP were significantly higher for the sperm from the JBC-A bull than for the sperm from the JBC-D bull (*p* < 0.05), but did not differ significantly between the sperm from the JCB-A bull and the sperm from the JBC-B, JBC-C, and JBC-E bulls.

The motility of frozen-thawed sperm (Table 2) ranged from 54.4% to 81.3% and was significantly lower than the motility of fresh sperm from the same bull. The frozen–thawed sperm from the JBC-A bull had significantly higher motility than the frozen–thawed sperm from the other four bulls (*p* < 0.05). VCL, VSL, and VAP were significantly higher for the sperm from the JBC-A bull than for the sperm from the JBC-B, JBC-D, and JBC-E bulls, but did not significantly differ between the sperm from the JBC-A bull and the sperm from the JBC-C bull. Overall, these findings show that the sperm from the JBC-A bull had the highest motility, whereas the sperm from the JBC-B bull had the lowest motility.

### 3.2. JBC Sperm Vitality

Sperm vitality was assessed using fresh and frozen–thawed sperm samples from the five bulls by eosin–nigrosin staining (Table 3). The vitality of fresh sperm from the JBC-A and JBC-E bulls was the highest and was significantly higher than that of fresh sperm from the JBC-B bull (*p* < 0.05). The vitality of frozen–thawed sperm from the JBC-A bull was significantly higher than that of frozen–thawed sperm from the JCB-B, JCB-C, and JBC-E bulls (*p* < 0.05). Overall, these findings show that sperm from the JBC-A bull had the highest vitality, whereas sperm from the JBC-B bull had the lowest vitality.

### 3.3. JBC Sperm Morphology

The morphology of fresh (Table 4) and frozen–thawed (Table 5) sperm from the individual bulls was assessed by Diff-Quik staining. Fresh sperm from the JBC-D bull had the highest percentage of morphologically normal spermatozoa, and this percentage was significantly higher than the corresponding percentage for fresh sperm from the JBC-E bull (*p* < 0.05).

Frozen–thawed sperm from the JBC-D bull had the highest percentage of normal spermatozoa, but this percentage did not significantly differ from the corresponding percentages for frozen–thawed sperm from the other bulls. An evaluation of abnormal sperm showed that the percentage of spermatozoa with a bent midpiece was higher than the percentages of spermatozoa with other abnormalities.

### 3.4. Pronuclear Formation of Bovine Oocytes In Vitro

**Figure 4:**
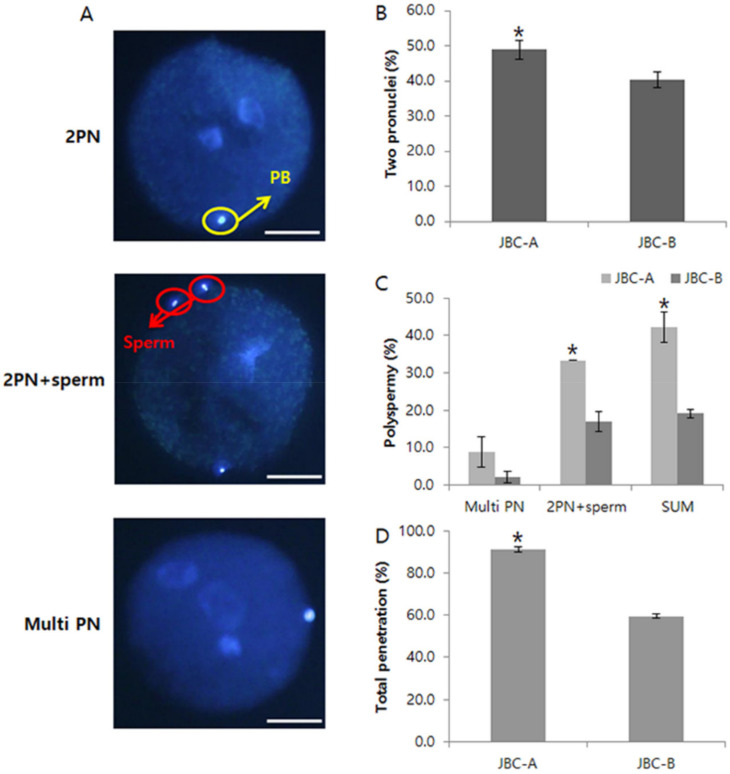
Nuclear status of bovine embryos at 18 h after in vitro fertilization. (**A**): Pronuclear status of bovine zygotes. (**B**): Percentage of zygotes with two pronuclei. (**C**): Percentage of zygotes with polyspermy. (**D**): Percentage of zygotes showing total penetration. Data shown are the mean ± SEM of three independent replicates per group. * *p* < 0.05. Scale bars = 30 μm.

The rates of polyspermy and formation of 2 PN at 18 h after insemination were analyzed (Figure 4). Sperm from the JBC-A bull had the highest rate of total penetration, and this rate was significantly higher than that of sperm from the JBC-B bull (*p* < 0.05). In addition, the rate of normal 2 PN formation was significantly higher for zygotes fertilized by sperm from the JBC-A bull than for zygotes fertilized by sperm from the JBC-B bull (*p* < 0.05). By contrast, the rate of polyspermy was significantly lower for zygotes fertilized by sperm from the JBC-B bull than for zygotes fertilized by sperm from the JBC-A bull.

## 4. Discussion

Little is currently known about the qualities of semen from JBC bulls, a breed of native Korean cattle living on Jeju Island. This breed is endangered because only about 1400 of these animals remain in Jeju Special Self-Governing Province. Elite breeding bulls are thus needed to preserve this breed and to further improve its traits. Spermatozoa must be sufficiently viable and motile for fertilization. The present study therefore evaluated the functional qualities of sperm samples from JBC bulls, including the motility, vitality, and morphology of spermatozoa, as the latter must be sufficiently viable and motile for successful fertilization. This analysis provided more precise information about the characteristics of sperm from individual JBC bulls and the use of these samples in assisted reproductive technologies (ARTs).

The present study was conducted to select the optimal JBC bulls for breeding. To determine the appropriate LN2 vapor exposure height for JBC bulls, experiments were conducted at heights of 3 cm and 7 cm, referring to 3 cm and 6.5 cm used in previous studies [12,13]. The motility of 3 and 7 cm sperm did not significantly differ for the JBC-B, JBC-C, JBC-D, and JBC-E bulls. However, the motility of 3 cm sperm (62.7 + 13.7%) was significantly lower than that of 7 cm sperm (81.3 + 2.9%) for the JBC-A bull (*p* < 0.05). These results suggest that JBC sperm were less damaged when they were frozen by exposure to LN2 vapor at a height of 7 cm and subsequent experiments were conducted based on these results.

The ability of mammalian spermatozoa to swim toward an oocyte is important for successful fertilization. Sperm propulsion is caused by the active motion of their flagella. Sperm quality has been assessed by measuring sperm motility, including measurements of VCL, VSL, and VAP [14,15,16,17,18]. The mean VCL, VSL, and VAP of sperm from Karan Fries bulls, a composite Indian breed, were found to be 188.63 ± 23.4 μm/s, 81.39 ± 16.2 μm/s, and 102.58 ± 20.5 μm/s, respectively [18]. The present study showed that the mean VCL, VSL, and VAP of frozen–thawed sperm from the JBC-A bull (93.5 ± 5.1 μm/s, 32.0 ± 1.5 μm/s, and 53.3 ± 2.7 μm/s, respectively) were higher than the corresponding measurements of frozen–thawed sperm from the four other JBC bulls. However, the mean motility of sperm from all JBC bulls was lower than that of sperm from Swiss Brown [14], Holstein-Friesian [17], and Karan Fries [18] bulls. These findings indicate that the motility of sperm from JBC bulls requires improvement.

Sperm vitality is estimated by assessing membrane integrity. The dye exclusion method is based on the principle that damaged plasma membranes, such as those found in non-vital (dead) cells, allow the entry of membrane-impermeant stains. In these assays, eosin is used to stain cells with damaged plasma membranes and nigrosin is used as a background stain [19]. The percentages of live and dead sperm allow the potential fertility of semen samples to be predicted because intact plasma membranes are required for fertilization [20]. Sperm with higher vitality are required to safely reach and fertilize oocytes, making sperm vitality a biological marker of sperm quality [16,21]. The present study showed that fresh sperm from the JBC-A and JBC-E bulls had the highest vitality. However, the freeze–thawing of sperm from the JBC-E bull significantly reduced its vitality. The vitality rates of semen from Brangus-Simmental cross-bred bulls ranged from 86.00% to 87.50% [21], whereas the vitality rate of sperm from mithun bulls was 54.96% [16]. By contrast, the vitality rate of frozen–thawed sperm from JBC bulls was much lower; it was 43.1 ± 3.2% for the JBC-A bull and even lower for the other four bulls. The World Health Organization has defined the lower limit of vitality for human semen as 58% [22], which is higher than that of frozen–thawed sperm from the JBC-A bull (43.1 ± 3.2%). Taken together, these results suggest that, of the five JBC bulls tested, sperm from the JBC-A bull had the highest vitality and motility, but that, in general, JBC sperm have lower vitality and motility than sperm from other breeds [18,21].

Fresh sperm from the JBC-D bull had the highest percentage of morphologically normal spermatozoa. After freeze–thawing, however, the percentage of normal sperm from this bull significantly decreased, whereas the percentages of sperm with abnormal heads and bent tails significantly increased. The percentage of human sperm with abnormal tails has also been found to increase after freeze–thawing [23]. The morphological normality of sperm is critical for sperm motility and successful fertilization. The morphological characteristics of sperm, including the morphologies of the sperm head, midpiece, and tail, not only reflect sperm quality and environmental factors during spermiogenesis, but are also important in the clinical diagnosis of male infertility [24]. Bulls with <60% normal sperm in the ejaculate have reduced fertility or are even infertile [25]. Our results showed that the proportion of normal sperm was less than 60% for each of the four JBC bulls, except the JBC-D bull, and therefore male fertility must be improved in this breed.

These analyses of the motility, vitality, and morphology of spermatozoa in fresh semen samples from five JBC bulls showed that sperm from the JBC-A, JBC-C, and JBC-E bulls were superior. After freeze–thawing, however, these parameters decreased in samples from the JBC-C and JBC-E bulls, suggesting that samples from these two bulls are more vulnerable to cryopreservation than samples from the JBC-A bull. Taken together, these results showed that motility and vitality were the highest for sperm from the JBC-A bull and lowest for sperm from the JBC-B bull, and these differences are reflected in the fertilization rates of semen samples from these two bulls.

Although the rate of normal 2 PN formation was the highest in zygotes fertilized with sperm from the JBC-A bull, the rate of polyspermy was also high. Monospermic fertilization occurs only when fully mature oocytes with a normal zona pellucida are fertilized with an optimal sperm concentration under optimized conditions [26]. An abnormal zona pellucida is one of the main causes of polyspermy in mammalian embryos [26]. In a previous study, the percentage of oocytes with polyspermy after culture for 20–24 h for maturation was 22%, whereas the percentage of oocytes with polyspermy after culture of fertilized oocytes for 44–48 h was 45% [27]. In addition, the failure of fertilization or cleavage shows that the development of oocytes derived from old cows is impaired at a very early stage [28], indicating that the developmental competence of oocytes decreases with maternal age [28]. Most bovine ovaries obtained from slaughterhouses in Jeju Special Self-Governing Province are from aged animals. The present results suggest that the superior vitality and motility of sperm from the JBC-A bull resulted in high rates of oocyte penetration and normal 2 PN formation. Although fertilization rates were high, so were the rates of polyspermy, largely due to the poor quality of oocytes. High-quality sperm and oocytes are needed to preserve and expand the population of endangered JBC.

## 5. Conclusions

The morphology, motility, vitality, and total penetration rate of sperm differed among samples obtained from five JBC bulls. Sperm morphology, motility, and vitality have long been regarded as good predictors of fertility in the absence of female infertility factors [29,30,31]. Our findings show that semen of the JBC-A bull may be superior to semen of the four other JBC bulls for the preservation and reproduction of this endangered breed using ARTs. The aging of the population of JBC breeding bulls suggests that a strategy must be devised to improve the generation of spermatozoa in vivo. The selection of JBC bulls for breeding requires the establishment of specifications to manage animals for high-quality semen production, and continuous monitoring will allow the mass reproduction of endangered JBC.

## Figures and Tables

**Table 1:** Motility parameters of fresh sperm from individual JBC bulls, as evaluated by SAIS.

Bull	Motility, %	VCL, μm/s	VSL, μm/s	VAP, μm/s	LIN, %	ALH, μm	STR, %	BCF, Hz
JBC-A	98.7 ± 0.8 ^a^	154.1 ± 9.0 ^a^	45.8 ± 2.4 ^a^	86.0 ± ±4.8 ^a^	31.4 ± 0.5	3.3 ± 0.2	53.9 ± 0.7	10.4 ± 0.5
JBC-B	96.1 ± 1.7 ^ab^	138.9 ± 5.5 ^ab^	41.0 ± 1.6 ^ab^	76.6 ± 3.0 ^ab^	31.3 ± 1.0	3.1 ± 0.1	53.6 ± 1.3	10.0 ± 0.2
JBC-C	96.1 ± 0.9 ^b^	139.4 ± 10.6 ^ab^	42.5 ± 2.6 ^ab^	78.5 ± 4.6 ^ab^	32.6 ± 1.3	3.1 ± 0.2	54.3 ± 1.4	9.7 ± 0.6
JBC-D	97.6 ± 2.0 ^ab^	130.9 ± 8.4 ^b^	38.2 ± 1.7 ^b^	71.9 ± 4.6 ^b^	31.5 ± 1.1	2.9 ± 0.2	53.4 ± 1.3	9.7 ± 0.4
JBC-E	97.4 ± 1.3 ^ab^	140.9 ± 7.6 ^ab^	43.3 ± 3.1 ^ab^	79.8 ± 4.4 ^ab^	32.2 ± 0.6	3.1 ± 0.1	54.0 ± 1.3	9.8 ± 0.4

Values represent the mean ± SEM of independent experiments. ^a,b^, *p* < 0.05. VCL, curvilinear velocity; VSL, straight-line velocity; VAP, average path velocity; LIN, linearity; ALH, amplitude of lateral head displacement; STR, straightness; BCF, beat cross frequency; JBC, Jeju black cattle.

**Table 2:** Motility parameters of frozen–thawed 7 cm sperm from individual JBC bulls, as evaluated by SAIS.

Bull	Motility, %	VCL, μm/s	VSL, μm/s	VAP, μm/s	LIN, %	ALH, μm	STR, %	BCF, Hz
JBC-A	81.3 ± 2.9 ^a^	93.5 ± 5.1 ^a^	32.0 ± 1.5 ^a^	53.3 ± 2.7 ^a^	37.9 ± 1.1 ^b^	2.1 ± 0.1 ^a^	60.2 ± 1.3	7.1 ± 0.3 ^a^
JBC-B	54.4 ± 5.9 ^b^	68.0 ± 4.7 ^b^	23.8 ± 1.1 ^b^	37.8 ± 2.6 ^b^	43.4 ± 1.6 ^a^	1.6 ± 0.1 ^b^	63.9 ± 1.7	4.5 ± 0.4 ^b^
JBC-C	66.0 ± 7.5 ^b^	80.1 ± 7.1 ^ab^	28.2 ± 2.0 ^ab^	45.6 ± 3.8 ^ab^	41.8 ± 3.0 ^ab^	1.8 ± 0.1 ^ab^	62.9 ± 3.1	5.6 ± 0.7 ^b^
JBC-D	58.7 ± 8.5 ^b^	70.2 ± 8.3 ^b^	25.0 ± 1.8 ^b^	41.3 ± 3.9 ^b^	40.1 ± 1.6 ^ab^	1.8 ± 0.1 ^b^	61.2 ± 1.6	5.4 ± 0.6 ^b^
JBC-E	64.9 ± 5.8 ^b^	76.4 ± 3.9 ^b^	26.3 ± 1.1 ^b^	43.2 ± 2.4 ^b^	39.8 ± 1.1 ^b^	1.8 ± 0.1 ^b^	61.2 ± 1.2	5.5 ± 0.3 ^b^

Values represent the mean ± SEM of independent experiments. ^a,b^, *p* < 0.05. VCL, curvilinear velocity; VSL, straight-line velocity; VAP, average path velocity; LIN, linearity; ALH, amplitude of lateral head displacement; STR, straightness; BCF, beat cross frequency; JBC, Jeju black cattle.

**Table 3:** Vitality of fresh and frozen–thawed 7 cm sperm from individual JBC bulls, as assessed by eosin–nigrosin staining.

Bull	Fresh, %	Frozen–Thawed, %
JBC-A	51.0 ± 3.9 ^a^	43.1 ± 3.2 ^a^
JBC-B	36.2 ± 8.4 ^b^	26.7 ± 5.0 ^b^
JBC-C	43.2 ± 6.9 ^ab^	30.4 ± 5.1 ^b^
JBC-D	44.3 ± 6.2 ^ab^	37.6 ± 4.2 ^ab^
JBC-E	51.6 ± 3.0 ^a^	31.5 ± 5.0 ^b^

Values represent the mean ± SEM of independent experiments. ^a,b^, *p* < 0.05. JBC, Jeju black cattle.

**Table 4:** Morphology of fresh sperm from individual JBC bulls, as assessed by Diff-Quik staining.

Bull	Normal, %	Abnormal Head, %	Detached Head, %	Abnormal Midpiece, %	Bent Midpiece, %	Bent Tail, %	Coiled Tail, %
JBC-A	58.9 ± 5.9 ^ab^	1.4 ± 0.3 ^b^	0.6 ± 0.1 ^b^	0.7 ± 0.3 ^ab^	31.2 ± 5.8 ^a^	6.1 ± 0.8 ^b^	1.1 ± 0.2
JBC-B	67.3 ± 4.4 ^ab^	1.3 ± 0.4 ^ab^	1.1 ± 0.3 ^ab^	0.9 ± 0.1 ^a^	17.9 ± ±3.7 ^b^	10.6 ± 2.0 ^a^	0.9 ± 0.2
JBC-C	70.7 ± 5.0 ^a^	2.3 ± 0.4 ^a^	0.9 ± 0.2 ^ab^	0.6 ± 0.4 ^ab^	17.7 ± 3.8 ^b^	7.0 ± 1.9 ^ab^	0.8 ± 0.3
JBC-D	71.1 ± 7.2 ^ab^	2.0 ± 0.3 ^ab^	0.6 ± 0.2 ^ab^	0.4 ± 0.1 ^b^	18.3 ± 5.0 ^ab^	7.1 ± 2.6 ^ab^	0.5 ± 0.3
JBC-E	58.5 ± 4.1 ^b^	1.3 ± 0.2 ^b^	1.5 ± 0.5 ^a^	1.2 ± 0.5 ^ab^	27.8 ± 3.7 ^a^	8.8 ± 1.9 ^ab^	0.9 ± 0.1

Values represent the mean ± SEM of independent experiments. ^a,b^, *p* < 0.05. JBC, Jeju black cattle.

**Table 5:** Morphology of frozen–thawed 7 cm sperm from individual JBC bulls, as assessed by Diff-Quik staining.

Bull	Normal, %	Abnormal Head, %	Detached Head, %	Abnormal MidPiece, %	Bent MidPiece, %	Bent Tail, %	Coiled Tail, %
JBC-A	52.4 ± 5.9	4.0 ± 0.6	0.6 ± 0.2	1.1 ± 0.4 ^ab^	23.4 ± 4.7 ^ab^	17.5 ± 1.5	1.0 ± 0.2
JBC-B	55.5 ± 4.8	3.6 ± 0.6	1.5 ± 0.6	0.6 ± 0.2 ^ab^	22.0 ± 3.6 ^ab^	15.7 ± 2.0	1.1 ± 0.4
JBC-C	52.2 ± 5.0	5.2 ± 1.1	0.8 ± 0.3	0.4 ± 0.2 ^ab^	25.9 ± 3.1 ^a^	13.7 ± 1.6	1.8 ± 0.5
JBC-D	61.8 ± 4.9	4.2 ± 0.6	0.6 ± 0.3	0.4 ± 0.1 ^b^	16.7 ± 3.2 ^b^	15.3 ± 3.0	1.0 ± 0.3
JBC-E	51.8 ± 3.5	3.3 ± 0.4	1.0 ± 0.3	1.0 ± 0.2 ^a^	26.2 ± 2.8 ^a^	15.3 ± 1.2	1.4 ± 0.5

Values represent the mean ± SEM of independent experiments. ^a,b^, *p* < 0.05. JBC, Jeju black cattle.

## Data Availability

The data that support the findings of this study are available upon reasonable request from the corresponding author (S.-P.P.).
